# ^2^H_2_O labeling methods for bulk and single muscle protein synthesis measures, and measures of integrated muscle protein synthesis and breakdown rates: A pilot study

**DOI:** 10.14814/phy2.71013

**Published:** 2026-07-09

**Authors:** Grith Højfeldt, Bjørk Wulff Helge, Gerrit van Hall, Peter Schjerling, Michael Kjær, Abigail L. Mackey, Jatin G. Burniston, Jakob Agergaard

**Affiliations:** ^1^ Department of Orthopedic Surgery, Institute of Sports Medicine Copenhagen Copenhagen University Hospital—Bispebjerg and Frederiksberg Copenhagen Denmark; ^2^ Department of Clinical Medicine, Faculty of Health and Medical Sciences University of Copenhagen Copenhagen Denmark; ^3^ Clinical Metabolomics Core Facility, Clinical Biochemistry, Rigshospitalet & Department of Biomedical Sciences, Faculty of Health & Medical Science University of Copenhagen Copenhagen Denmark; ^4^ Research Institute for Sport and Exercise Sciences Liverpool John Moores University Liverpool UK

**Keywords:** D_2_O, dynamic proteome profiling, muscle protein breakdown, muscle protein synthesis

## Abstract

This methodological pilot study explored the feasibility of an integrated tracer approach for simultaneous measurement of human skeletal muscle protein synthesis and breakdown during prolonged deuterium oxide (^2^H_2_O) labeling. Four healthy men completed a five‐week protocol combining daily oral ^2^H_2_O intake with selected stable isotope amino acid tracers to assess bulk fractional synthesis rates, fractional breakdown rates, and single‐protein turnover. The study specifically addressed the methodological question of whether bulk or individual protein synthesis measurements remain consistent over a prolonged ^2^H_2_O labeling period. We suggest that measurements of mixed‐protein fractions in muscle might be vulnerable to error due to the rise‐to‐plateau kinetics associated with longer‐duration labeling. Mean mixed‐protein synthesis rates could be underestimated when labeling exceeds approximately 4 weeks. Increasing precursor enrichment mitigated this effect but highlights the need for controlled enrichment strategies in long‐term studies. In contrast, dynamic proteome profiling (DPP) provided more robust single‐protein turnover estimates across the full period because the different isotopic plateaus of individual peptides could be modeled. The study also tested the feasibility of combining ^2^H_2_O labeling with ^15^N‐alanine–based breakdown measurements and evaluated potential interference from multiple tracers on DPP outputs. Together, these findings outline practical considerations, limitations, and feasibility of an integrated multi‐tracer framework for studying human muscle protein turnover over extended periods.

## 1 INTRODUCTION

Tissues of the body exist in a dynamic state of renewal, defined by the balance between the synthesis and breakdown of their constituent proteins. Shifts in the balance between protein synthesis and breakdown allow tissues to adapt, for example, during development or in response to environmental changes, such as exercise, injury recovery, disuse, disease, and the natural process of aging.

Significant insights into acute changes in human muscle protein synthesis, for example in the hours after a protein‐rich meal or a bout of resistance exercise (Rennie, 1999; Rennie & Tipton, 2000; Rasmussen et al., 2000), have been provided by studies using short term infusions of stable isotope‐labeled amino acids to trace amino acid incorporation rate into tissue proteins. However, it is challenging to study longer‐term adaptations using methodologies that require intravenous infusion. Deuterium oxide (^2^H_2_O, also known as ‘heavy water’), can be delivered via a participant's drinking water over periods of days or weeks to study protein turnover integrated across daily life. Deuterium oxide rapidly distributes in the body water compartment and equilibrates into amino acids through transamination reactions. Alanine, with a high turnover, becomes rapidly enriched with ^2^H (Previs et al., 2004) and, therefore, has become the preferred target for isotope‐ratio mass spectrometry measurements on deuterium incorporation into tissue proteins. In recent years, studies utilizing deuterium oxide have brought significant new insight into the longer‐term responses of human muscle to experimental interventions such as resistance exercise training (Brook et al., 2015) or immobilization (Mitchell et al., 2018).

The transition from shorter duration studies employing amino acid tracers to longer duration studies using deuterium oxide involves a number of technical considerations, which have not yet been fully addressed in the literature. For example, short‐term studies rely on precursor‐product calculations, which anticipate a linear relationship between protein synthesis and the incorporation of label into protein to calculate protein fractional synthesis rates. In contrast, longer‐term investigations using deuterium oxide labeling exhibit rise‐to‐plateau kinetics, necessitating the use of non‐linear models (Busch et al., 2006). At the onset of a labeling period, label is incorporated into protein relatively rapidly before approaching a plateau where a balance is reached between the probability that synthesis will add label to the protein pool (i.e. dictated by precursor enrichment) and degradation will remove label from the protein pool (i.e. assuming degradation is a stochastic process with equal preference for labeled and unlabelled protein). It is speculated that, in humans, labeling periods longer than 4 weeks could be sufficient for the incorporation of deuterium into muscle protein to plateau, which would lead to an underestimation of protein turnover rates (Holwerda et al., 2024). However, as yet, there is no empirical evidence to support this proposition and the period that avoids a plateau during bulk muscle protein synthesis measurements is unknown. Furthermore, rise‐to‐plateau kinetics may not be evident from the analysis of muscle fractions that contain mixtures of proteins each with different synthesis rates (Miller et al., 2015) and protein‐specific measurements are likely required to explored whether longer periods of labeling affect the outcome of protein synthesis measurements.

Muscle fractions such as sarcoplasmic, mitochondrial, myofibrillar, or connective tissue each contain hundreds of different proteins, which may change turnover rate and/or abundance in response to exercise training (Camera et al., 2017; Srisawat et al., 2024). Getting pure fractions with current methods can be challenging (Holwerda et al., 2025). Furthermore, processes such as muscle transformation or tissue regeneration do not merely change the turnover or abundance of existing proteins, but also involve the synthesis and degradation of entirely different proteins at different stages (Hesketh et al., 2020; Højfeldt et al., 2023). Such protein‐specific changes are hidden from measurements at the amino acid level where the link between the rate of isotope incorporation and identity of the protein is lost. The dynamic proteome profiling (DPP) technique (Camera et al., 2017) uses deuterium oxide labeling and peptide mass spectrometry to simultaneously measure both the abundance and rate of synthesis of proteins in vivo in human muscle (Camera et al., 2017; Srisawat et al., 2020, 2024) as well as rodents (Hesketh et al., 2020; Holwerda et al., 2020; Stead et al., 2025). In a mouse study, which explored skeletal muscle regeneration, there was a clear distinction in the protein turnover profiles between the different phases of regeneration emphasizing the importance of single protein analysis (Bizieff et al., 2024). In proteins with high turnover rates, a plateau in protein enrichment may occur within a relatively short time span, making it particularly important to consider the duration of the labeling period when doing DPP. Furthermore, studies are often designed with the application of multiple isotope tracers with the aim of exploring protein synthesis (e.g., with ^2^H_2_O labeling) and breakdown simultaneously, and to study whole body protein metabolism (Højfeldt et al., 2021; Justesen et al., 2022). In such a multiple‐tracer scenario, it is unknown whether peptide analysis using the DPP method is affected by isotopes other than the deuterium labeling from ^2^H_2_O administration.

If amino acid tracers are infused around the time of the baseline sample collection in DPP studies, ^15^N‐labeled amino acids could alter the mass isotopomer pattern of peptides in a manner that is similar to the effect of deuterium incorporation. Whereas, the incorporation of a label such as ^2^H_8_‐phenylalanine would increase the mass/charge ratio of phenylalanine‐containing peptides by 8 Thompsons which would effectively split the signal across two or more peptide envelopes, that is, in a manner similar to SILAC analyses in cells (Ong et al., 2002). Potentially, each of these scenarios could confound the calculation of protein synthesis rates in DPP studies.

Whole body or arteriovenous (AV) balance measurements allow for the simultaneous assessment of protein synthesis and breakdown. From these types of studies, we know how exercise, nutrition, disuse, and disease acutely influence net protein balance at both the whole body and mixed muscle protein levels (Agergaard et al., 2023; Biolo et al., 1997; Brook et al., 2022; Hirsch et al., 2020; Højfeldt et al., 2020; Hudson et al., 2022; Phillips et al., 1997). Muscle‐specific protein breakdown becomes particularly important in conditions such as diseases with high amino acid demand, tissue regeneration involving extensive protein remodeling, or combined exercise and nutrition interventions aimed at enhancing net protein balance (Tipton et al., 2018; Wolfe, 2006). In these cases, shifting from acute to integrated measurements of muscle protein breakdown is warranted. Integrated measurements of muscle protein breakdown with preceding ^2^H_2_O labeling have been demonstrated (Dideriksen et al., 2020; Holm et al., 2013). Also, simultaneous measurements of integrated muscle protein synthesis and breakdown have been attempted, albeit breakdown measurements with methyl[D_3_]‐methylhistidine were limited to a 6‐h measurement (Cegielski et al., 2021). Therefore, improved methods for simultaneous measuring integrated muscle protein synthesis and breakdown are necessary.

With the goal to simultaneously measure integrated muscle protein synthesis and breakdown (i.e. protein turnover), and to combine the application of infused stable‐isotope amino acid tracers with dynamic proteome profiling of single protein synthesis, we set up a pilot study. The study aimed to investigate whether bulk or individual protein synthesis measurements remain consistent over a prolonged ^2^H_2_O labeling period. It also sought to explore the feasibility of simultaneously measuring integrated muscle protein synthesis and muscle protein breakdown, and to assess whether multiple tracer labeling impacts the analysis of single muscle protein synthesis.

## 2 METHODS

### 2.1 Participants

Four healthy male participants aged 18–35 years, with a BMI between 18 and 30 kg/m^2^, and with normal and regular diet habits were recruited through web‐based advertisement for the study. Exclusion criteria included elevated blood pressure, abnormal resting heart rate, clinically abnormal blood values, smoking, engagement in regular strength training within 3 months prior to the trial, a history of anabolic steroid or growth hormone use, corticosteroid use in the last 3 months, current use of blood‐thinning medication or medication affecting muscle protein turnover, medical or surgical conditions potentially impacting protein turnover or trial participation, a history of substance or alcohol abuse, or previous participation in trials involving ^2^H_2_O or amino acid tracers. Inclusion interviews were conducted to ensure that the participants met the inclusion and exclusion criteria. All participants provided written consent prior to enrolling in the study, in line with the Declaration of Helsinki. The Ethics Committee of Copenhagen and Frederiksberg (H‐21025129) approved the study before the project began. If included in the study, whole body dual‐energy x‐ray absorptiometry (DXA) scans were performed to determine lean body mass (LBM).

### 2.2 Study design

The present study comprised a pilot test with the aim to investigate a model for simultaneous measurement of integrated muscle protein synthesis and muscle protein breakdown. Furthermore, the aim was to investigate confounding factors, such as the labeling period of deuterium oxide (^2^H_2_O), on the measurement of muscle protein synthesis on a bulk protein (muscle fractions) or single protein level.

**FIGURE 1:**
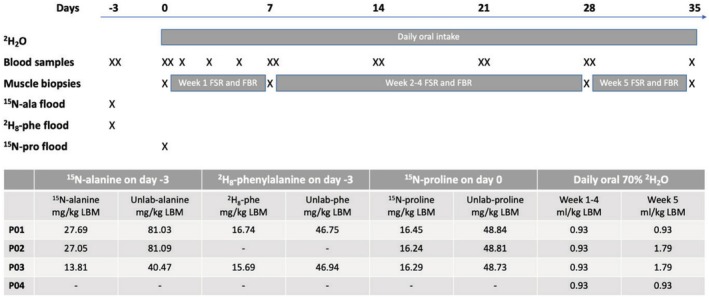
Study design and tracer administration. Study design with indication of time points for blood and muscle tissue sampling and time points for AA‐tracer floods. Table is showing for Participants P01, P02, P03, and P04 the weight of the stable isotope‐labeled amino acids and the unlabeled amino acids infused on Day −3 and Day 0, and daily oral ^2^H_2_O administration during the study after the initial loading days.

The participants underwent a five‐week measurement period (Figure 1). The specific tracer administration was individualized as described later. In general, 3 days before the measurement period began, participants received tracer floods with ^15^N‐alanine and ^2^H_8_‐phenylalanine. From the beginning of the measurement period, participants daily received oral ^2^H_2_O. Blood samples were drawn throughout the study, and muscle tissue biopsies were taken before commencing the five‐week period and after 1, 4, and 5 weeks.

The participants were instructed to maintain their usual diet and physical activity habits over the five‐week period. None of them engaged in regular or structured exercise. Generally, their physical activity consisted of light exercise, primarily involving cycling as a means of daily transportation to and from work or school.

### 2.3 Stable isotope tracer design

#### 2.3.1 ^2^H_2_O administration

Deuterium oxide (^2^H_2_O, DLM‐4‐0, Cambridge Isotope Laboratories Inc., MA, USA) was administered orally to obtain ^2^H‐alanine precursor enrichment used for protein synthesis measurements on fractions of sarcoplasmic, myofibrillar, and connective tissue proteins, as well as single protein synthesis using dynamic proteome profiling.

Participants received ^2^H_2_O with a dose aiming at 1% body water enrichment, except for Week 5 where two participants (P02 and P03) increased the ^2^H_2_O dose aiming at an increase to 2% body water enrichment (Figure 1). The ^2^H_2_O loading was commenced with three loading days with 7.14 mL of 70% ^2^H_2_O/kg LBM distributed in 5 boluses interspersed by 1 h on Day 0, 4.29 mL of 70% ^2^H_2_O/kg LBM distributed in 3 boluses interspersed by 1 h on Day 1, and 1.43 mL of 70% ^2^H_2_O/kg LBM distributed in 1 bolus on Day 2. On subsequent days, 0.93 mL of 70% ^2^H_2_O/kg LBM was administered in 1 daily bolus.

On Day 28, the ^2^H_2_O dosage was increased for participants P02 and P03 to achieve 2% body water enrichment. This was commenced by 7.14 mL of 70% ^2^H_2_O/kg LBM distributed in 5 boluses interspersed by 1 h on Day 28, 5.00 mL of 70% ^2^H_2_O/kg LBM distributed in 4 boluses interspersed by 1 h on Day 29, and 2.86 mL of 70% ^2^H_2_O/kg LBM distributed in 2 boluses interspersed by 1 h on Day 30. On subsequent days, 1.79 mL of 70% ^2^H_2_O/kg LBM was administered in 1 daily bolus.

On Days 0, 1, 7, 14, 21, 28, and 29 all ^2^H_2_O intake was closely monitored by the research staff. On all other days, the ^2^H_2_O intake was performed at home and monitored by research staff through daily questionnaires. At each visit (Days 0, 7, 14, 21, and 28), the participants received bottles for the following week labeled with weekday and filled with the correct volume of ^2^H_2_O.

The loading days (Days 0, 1, and 2, and 28, 29, and 30 for participant P02 and P03) were designed to minimize the risk for side effects from ^2^H_2_O intake, normally nausea and vertigo. Only mild nausea was experienced for some of the participants on Days 0 and 1, which quickly disappeared.

#### 2.3.2 Tracer infusions

To measure muscle protein breakdown and potential confounding factors from tracer administration on single DPP analysis, stable isotope‐labeled amino acid tracer floods of ^15^N‐alanine (NLM‐454‐MPT‐PK, Cambridge Isotope Laboratories Inc., MA, USA), ^2^H_8_‐phenylalanine (DLM‐372‐MPT‐PK, Cambridge Isotope Laboratories Inc., MA, USA), and ^15^N‐proline (NLM‐835‐MPT‐PK, Cambridge Isotope Laboratories Inc., MA, USA) were given. Participant P04, who did not receive any intravenous tracer infusions, served as a control. The tracers were infused through a venous catheter placed in an antecubital vein. In sterile saline, each tracer was dissolved at a 1:4 w/w ratio with its respective unlabeled amino acid: unlabeled‐alanine (L‐alanine, 1.01700, Merck, Germany), unlabeled‐phenylalanine (ULM‐8205‐PK, Cambridge Isotope Laboratories Inc., MA, USA), and unlabeled‐proline (ULM‐8333‐MPT‐PK, Cambridge Isotope Laboratories Inc., MA, USA).

On Day −3, participants P01 and P02 received ^15^N‐alanine at a dose of 300 μmol/kg LBM, while participant P03 received a half‐dose of 150 μmol/kg LBM. This tracer was used to assess muscle protein breakdown. ^2^H_8_‐phenylalanine was administered to participants P01 and P03 at 90 μmol/kg LBM on Day −3 as a negative control to evaluate the potential re‐circulation and reincorporation of the tracer. On Day 0, ^15^N‐proline was administered to participants P01, P02, and P03 at a dose of 140 μmol/kg LBM to study collagen turnover in tendon samples (not included in this paper) and to examine any interference with the DPP analysis. The measured tracer weights for each participant are shown in Figure 1.

### 2.4 Precursor measurements

#### 2.4.1 Blood sampling

Blood samples were collected from the antecubital veins on Days −3, 0, 1, 3, 5, 7, 14, 21, 28, 29, and 35 to measure plasma tracer enrichments. On Day −3, plasma samples were taken before and 30 min after the tracer flood. On Days 0 and 28 (for participants P02 and P03 only), blood samples were drawn before the first bolus and 2 h after the last bolus of ^2^H_2_O. On Days 7, 14, and 21, blood samples were collected before and 2 h after a single bolus of ^2^H_2_O. Finally, on Day 35, a plasma sample was taken before the muscle biopsy.

The blood samples were collected in K3‐EDTA vacutainer tubes. After collection, the tubes were placed on ice for 10–30 min. Subsequently, the samples were centrifuged at 3200*g* for 10 min at 4°C to separate the plasma. The plasma was then aliquoted into 500 mL tubes and stored at −80°C until further analysis.

#### 2.4.2 Mass spectrometry

For the mass spectrometry analysis of plasma amino acid tracer precursor enrichment, 200 mL of plasma per sample was used to extract free amino acids. Isotopically labeled internal standards (uniformly labeled with ^13^C/^15^N) for each amino acid were added to the plasma in a 50% acetic acid solution. The plasma solution was then poured over cation exchange columns containing AG 50 W‐X8 resin (Bio‐Rad Laboratories), which had been prepared with 3 × 2 mL of 1 M HCl. The resin columns were washed five times with 3 mL of deionized water before eluting the amino acids into collection vials by adding 2 × 2 mL of 4 M NH_4_OH. To measure plasma amino acid tracer enrichment, the solvents eluted from the resin columns were evaporated under a stream of N_2_ at 70°C. The samples were then derivatized into their PITC‐derivative using phenylisothiocyanate (PITC) (P1034‐10x1ML, Merck, Germany). Ten microliters of the derivatized samples were loaded and analyzed by LC–MS/MS (TSQ Quantiva; Thermo Fisher Scientific, San Jose, CA) as described elsewhere (Bornø & Van Hall, 2014).

#### 2.4.3 Tissue sampling

Muscle biopsies were conducted on Days 0, 7, 21, and 35 from the vastus lateralis muscle. These biopsies were taken in a randomized sequence from four different incisions, each spaced 3 cm apart. The muscle biopsies were performed under local anesthesia using 1% lidocaine. The samples were collected with 4‐mm Bergström biopsy needles (Stille, Stockholm, Sweden) using manual suction. Blood and visible fat were promptly removed from the muscle specimens, which were then rinsed with saline water, snap‐frozen in liquid nitrogen, and stored at −80°C until further analysis.

### 2.5 Sample preparation for bulk myofibrillar, sarcoplasmic, and connective tissue

#### 2.5.1 Homogenization of muscle tissue

Approximately 20 mg of muscle tissue was placed in a 2 mL vial with a screw cap, containing 8 beads and 2 silicon crystals. One milliliter of homogenization buffer (Tris 0.02 M [pH 7.4], NaCl 0.15 M, EDTA 2 mM, EGTA 2 mM, TritonX‐100 0.5%, sucrose 0.25 M) was added. The tissue was homogenized using a FastPrep (Thermo Savant, Holbrook, NY, USA) at speed 5.5 for 4 cycles of 45 s each, with a 2‐min pause between cycles. The homogenate was then incubated at 5°C for 3 h and subsequently centrifuged at 1600*g* for 20 min at 5°C.

#### 2.5.2 Sarcoplasmic protein extraction

The supernatant was carefully transferred to a new 2 mL vial with a screw cap, ensuring the pellet was not disturbed. Proteins were precipitated by adding 2.36 volumes of ice‐cold (−20°C) 99% ethanol, followed by immediate mixing by inversion. The sample was centrifuged at 1600*g* for 20 min at 5°C, and the supernatant was discarded. The resulting pellet, containing the sarcoplasmic protein fraction, was hydrolysed by adding 1 mL of 6 M HCl and incubating overnight at 110°C.

#### 2.5.3 Myofibrillar and connective tissue protein extraction

The remaining pellet from the sarcoplasmic protein extraction was resuspended in 1.0 mL of homogenization buffer and homogenized for 45 s at speed 5.5 m/s using the FastPrep. The sample was incubated at 5°C for 30 min and centrifuged at 1600*g* for 20 min at 5°C. The supernatant was discarded, and the pellet was resuspended in 1.5 mL of KCl buffer (KCl 0.7 M, pyrophosphate (Na4P2O7) 0.1 M). The sample was vortexed vigorously to ensure complete resuspension and incubated overnight at 5°C. The following day, the sample was vortexed and centrifuged at 1600*g* for 20 min at 5°C.

#### 2.5.4 Myofibrillar protein fraction

The supernatant was transferred to a new 2 mL vial with a screw cap. Proteins were precipitated by adding 2.3 volumes of ice‐cold (−20°C) 99% ethanol, followed by immediate mixing by inversion. The sample was incubated at 5°C for 2 h and centrifuged at 1600*g* for 20 min at 5°C. The supernatant was discarded, and the pellet was resuspended in 1 mL of 70% ethanol, vortexed, and centrifuged again at 1600*g* for 20 min at 5°C. The supernatant was discarded, and the pellet, containing the myofibrillar protein fraction, was hydrolyzed by adding 1 mL of 6 M HCl and incubating overnight at 110°C.

#### 2.5.5 Connective tissue protein fraction

The remaining pellet from the myofibrillar protein extraction was resuspended in 1 mL of KCl buffer to wash the pellet. The sample was vortexed vigorously to ensure complete resuspension and incubated at 5°C for 2 h. The sample was centrifuged at 1600*g* for 20 min at 5°C, and the supernatant was discarded. The pellet was resuspended in 1 mL of 70% ethanol, vortexed, and incubated at 5°C for 30 min. The sample was centrifuged at 1600*g* for 20 min at 5°C, and the supernatant was discarded. The pellet, containing the connective tissue protein fraction, was hydrolysed by adding 1 mL of 6 M HCl and incubating overnight at 110°C.

### 2.6 Mass spectrometry

The amino acids of the hydrolysed solutions of sarcoplasmic, myofibrillar, and connective tissue fractions were extracted on resin columns as described for plasma samples. The samples were then derivatized to convert the amino acids into their NAP derivatives. Of each sample, 2 μL were analyzed by GC‐C‐IRMS (Delta V, Thermo Fisher Scientific, San Jose, CA) as detailed elsewhere (Bornø et al., 2014).

### 2.7 Equations

Herein we use both the traditional term fractional synthesis rate as well as fractional turnover rate (FTR) dependent on the situation. FTR which is a more suitable term than FSR when the period of isotope labeling is relatively long. Under such conditions, the rate of incorporation of label into a protein is modeled by non‐linear equations that reflect the combined processes of synthesis and degradation, that is, protein turnover. In the current work we have studied human muscle under steady state conditions of precursor enrichment and protein abundance. Under such constraints, the incorporation of deuterium into protein can be modeled as a rise‐to‐plateau (asymptotic regression) in relative isotopomer abundance (RIA) (Equation 4) that reflects the contributions of both synthesis and degradation to the replacement (i.e. turnover) of the protein pool (Burniston, 2019). At the onset of the labeling period, the probability that a newly synthesized protein will include deuterium label is set by the level of precursor enrichment, whereas protein degradation is likely to remove unlabelled protein, that is, given that the large existing pool of protein is unlabelled. Therefore, label is incorporated into protein relatively rapidly before reaching a plateau as a balance is neared between the probability that synthesis will add label to the protein pool (i.e. dictated by precursor enrichment) and degradation will remove label from the protein pool (i.e. assuming degradation is a stochastic process with equal preference for labeled and unlabelled protein) (Burniston, 2019).

Fractional Synthesis Rate (FSR) and Fractional Breakdown Rate (FBR) were measured at Days 0–7, Days 7–28, and Days 28–35. The fractional synthesis rates (FSR) of sarcoplasmic, myofibrillar, and connective tissue muscle proteins were calculated based on direct incorporation of the orally ingested ^2^H_2_O derived ^2^H‐alanine:

(1) $$\mathrm{FSR}=\left[\frac{∆E_{\text{protein}}}{{\hat{E}}_{\text{precusor}}\times ∆t}\right]\times 100$$

where FSR denotes the fractional synthetic rate in % × day^−1^. Δ*E*_protein_ is the change in muscle protein enrichment of ^2^H‐alanine from the first biopsy to the last biopsy within the measurement interval, the time interval which defines Δ*t* in days, and the time period over which the weighted average precursor enrichment (*Ê*_precursor_) of ^2^H‐alanine was measured in plasma. Importantly, the *Ê*_precursor_ was calculated as a weighted average that accounts for potential variability in precursor enrichment because it gives more influence on the enrichment values that contributed more to tracer incorporation over time.

The fractional breakdown rates (FBR) of sarcoplasmic, myofibrillar, and connective tissue muscle proteins were calculated based on protein disappearance of the pre‐labeled ^15^N‐alanine tracer:

(2) $$\mathrm{FBR}=\left[\frac{-\ln\left(\frac{E_{x}}{E_{0}}\right)}{∆t}\right]\times 100$$

where FBR denotes the fractional breakdown rate in % × days^−1^. *E*_*x*_ is the muscle protein bound tracer enrichment at final time point *x*, *E*_0_ is the muscle protein bound tracer enrichment at initial time point 0, and Δ*t* is the time difference in days between the time point *t*_*0*_ for the initial muscle biopsy and the time point *t*_x_ for the final muscle biopsy.

### 2.8 Dynamic proteome profiling

#### 2.8.1 Sample preparation

Muscle tissue samples were lysed with lysis buffer (1% (w/v) Sodium Deoxycholate, 100 mM TEAB, pH 8.5) in a 96‐well BeatBox tissue homogenizer (Preomics) for 10 min at standard setting, followed by incubation for 10 min at 95°C. BeatBox homogenization and boiling were repeated for a total of five rounds. TCEP (Thermo Fisher Scientific, CAT #77720) was added to 5 mM, and CAA (Sigma Aldrich, CAT #22790‐250G‐F) to 20 mM, and incubated at 45°C for 20 min. Samples were digested by addition of Trypsin (Seq. Gr. Mod, Trypsin, bulk scale, Promega, CAT # V511X) and Lys‐C (Lysine C, Fujifilm, CAT #129–02541) at a 1:100 enzyme/protein ratio. Peptides were desalted using iST plates (iST Positive Pressure Plate 96×, Preomics, CAT # SFG00084), dried down, and resuspended in buffer A* (2% ACN, 0.1% TFA) for LC–MS analysis.

#### 2.8.2 LC–MS (DDA)

Peptides were separated on a 50 cm uPAC Gen3 column with a Vanquish Neo HPLC system (Thermo Fisher Scientific) coupled through a nano‐electrospray source to a Tribrid Eclipse mass spectrometer (Thermo Fisher Scientific) with a non‐linear gradient of 4%–45% buffer B (0.1% formic acid, 80% acetonitrile) at a flow rate of 250 nL/min over 90 min. The column temperature was kept at 50°C. Samples were acquired using a DDA MS2 data acquisition, where the Tribrid mass spectrometer was switching between a full scan (120 K resolution, 50 ms max. injection time, AGC target 100%) to a data‐dependent (Top‐20) MS/MS scans in the Orbitrap analyzer (15 K resolution, 22 ms max. injection time, AGC target 200%). The isolation window was set to 1.4 (m/z), and normalized collision energy to 30. Precursors were filtered by charge state of 2–6 and multiple sequencing of peptides was minimized by excluding the selected peptide candidates for 60 s.

#### 2.8.3 Label‐free quantitation of protein abundances

Protein abundances were measured by label‐free quantitation using Progenesis Quantitative Informatics for Proteomics (QI‐P; Nonlinear Dynamics, Waters Corp., Newcastle, UK, Version 4.2), consistent with previous studies (Burniston, 2019; Burniston et al., 2014; Camera et al., 2017; Hesketh et al., 2020). Log‐transformed MS data were normalized by inter‐sample abundance ratio, and relative protein abundances were calculated using nonconflicting peptides only. MS/MS spectra were exported in Mascot generic format and searched against the Swiss‐Prot database (2021_03) restricted to Homo‐sapiens (20,371 sequences) using a locally implemented Mascot server (v.2.8; www.matrixscience.com). The enzyme specificity was trypsin with 2 allowed missed cleavages, carbamidomethylation of cysteine (fixed modification) and oxidation of methionine (variable modification). MS data were searched with m/z error tolerances of 10 ppm for peptide ions and 0.6 Da for fragment ion spectra. Peptide results were filtered to 1% FDR based on decoy search, and at least 1 unique peptide was required to identify each protein. The Mascot output (xml format), restricted to non‐homologous protein identifications, was recombined with MS profile data in Progenesis.

#### 2.8.4 Measurement of protein synthesis rates

Mass isotopomer abundance data were extracted from MS spectra using Progenesis Quantitative Informatics (Non‐Linear Dynamics, Newcastle, UK). Consistent with previous work (Camera et al., 2017; Hesketh et al., 2020; Stansfield et al., 2021), the abundances of the monoisotopic peak (m_0_), m_1_, m_2_, and m_3_ mass isotopomers were collected over the entire chromatographic peak for each proteotypic peptide that was used for label‐free quantitation of protein abundances. Mass isotopomer information was processed using in‐house scripts written in Python (version 3.12.4). Incorporation of ^2^H_2_O into newly synthesized protein results in an increase in the relative isotopomer abundance (RIA) of the m_1_ mass isotopomer relative to the sum of the m_0_ and m_1_ mass isotopomers that follows rise‐to‐plateau kinetics of an exponential regression (Sadygov, 2020).

(3) $$\mathrm{RIA}=\frac{m_{1}}{m_{0}+m_{1}}$$

The rate constant of protein degradation (*k*_deg_) was calculated from the rise in RIA during each labeling period based on exponential regression (rise‐to‐plateau) kinetics. The plateau in RIA (RIA_plateau_) of each peptide was derived from the total number (N) of ^2^H exchangeable H—C bonds in each peptide, which was referenced from standard tables (Holmes et al., 2015) and the difference in the D:H ratio (^2^H/^1^H) between the natural environment (DH_nat_) and the experimental environment (DH_exp_) based on the molar percent enrichment of deuterium in the precursor pool, according to (Sadygov, 2020)

(4) $${\mathrm{RIA}}_{\text{plateau}}=1-\left(\frac{1}{\left(\frac{1}{1-\mathrm{RIA}\left(t_{0}\right)}\right)+N\left({\mathrm{DH}}_{\exp}-{\mathrm{DH}}_{\mathrm{nat}}\right)}\right)$$

The rate constant of protein degradation (*k*_deg_) was calculated either by non‐linear least squares regression across samples collected at Days 0, 7, 28 and 35 or by 2‐point calculations (Equation 5) between the beginning (*t*_0_) and end (*t*_1_) of each 7‐day labeling period, that is, Day 0–Day 7 or Day 28–Day 35. Calculations for exponential regression (rise‐to‐plateau) kinetics reported in (Sadygov, 2020) were used and *k*_deg_ data were adjusted for differences in protein abundance (P) between the beginning (*t*_0_) and end (*t*_1_) of each labeling period.

(5) $$k_{\deg}=-\frac{1}{t-t_{0}}•\ln\left(1-\frac{\mathrm{RIA}\left(t_{1}\right)-\mathrm{RIA}\left(t_{0}\right)}{\;{\mathrm{RIA}}_{\text{plateau}}-\mathrm{RIA}\left(t_{0}\right)}\;\right)⦁\frac{\;P\left(t\right)}{P\left(t_{0}\right)}$$

Fractional turnover rates (FTR; %/d) were derived by multiplying decimal *k*_deg_ values by 100 and weighted FTR values were calculated by multiplying FTR by protein abundance normalized to the total abundance of all proteins included in the analysis.

### 2.9 Data presentation/statistics

This pilot study investigates the tracer setup for simultaneously measuring muscle protein synthesis and breakdown, while also examining confounding factors affecting single protein synthesis measurements. Given the different tracer setups for each of the four participants, no statistical tests were conducted. Instead, the data are evaluated and presented as individual values. Data illustrations and descriptive statistical analyses were performed in R version 4.4.0. Pearson's correlation was used to assess similarities in proteome profile amongst the baseline samples. Non‐linear least squares regression with Levenberg–Marquardt algorithm was conducted using the *R* package minpack.lm (version 1.2–4), and self‐starting parameters, including: (i) the predicted asymptotic plateau, (ii) baseline starting value of the unlabelled peptide and (iii) an estimated rate constant of 0.01.

## 3 RESULTS

### 3.1 Plasma precursor enrichments of ^2^H‐alanine

**FIGURE 2:**
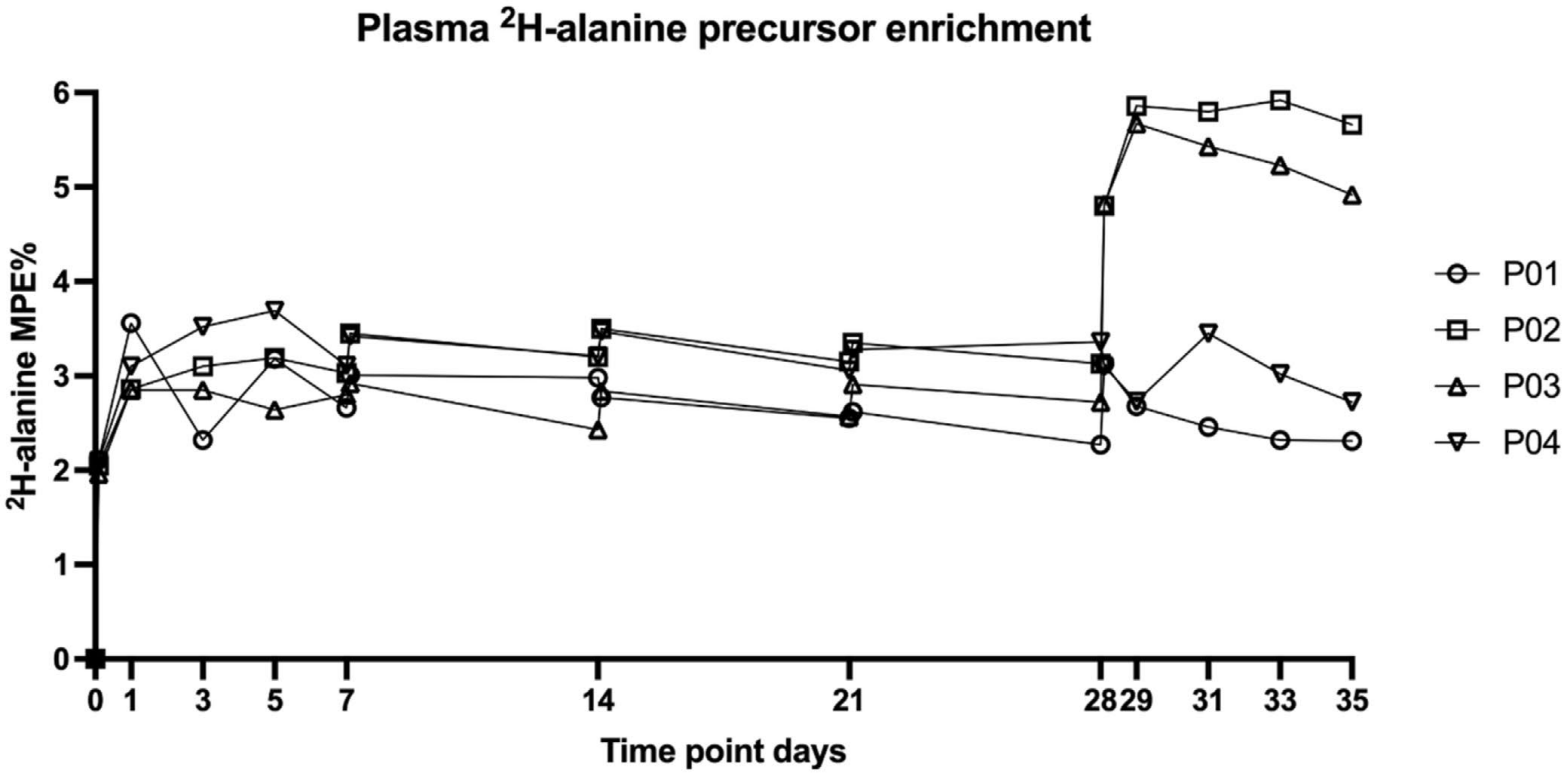
Plasma ^2^H‐alanine precursor enrichment. ^2^H_2_O administration was commenced on Day 0 for Participants P01, P02, P03, and P04. Daily intake of ^2^H_2_O maintained the ^2^H‐alanine precursor enrichment. From Day 28 to Day 35, Participants P02 and P03 increased the ^2^H_2_O intake to double the level of precursor enrichment. The background ^2^H‐alanine enrichment is subtracted from the precursor enrichment and expressed as mole percent excess (MPE).

When the oral ^2^H_2_O administration was initiated on Day 0, an immediate increase in plasma ^2^H‐alanine enrichment was seen for all participants and maintained a steady state from Day 1 to Day 28 (Figure 2). From Day 28 to Day 35, the two participants (P02 and P03) who ingested a greater dose of ^2^H_2_O also achieved an immediate and clear increase in plasma ^2^H‐alanine enrichment. By this, participants P02 and P03 clearly separated from the two participants (P01 and P04) who maintained a consistent dose and therefore a steady state plasma ^2^H‐alanine enrichment throughout the entire 35‐day period. It was observed that participant P01 had the greatest fluctuations in plasma ^2^H‐alanine enrichment in the initial 7 days. Also, during the last 14 days of the study, participant P01 had the lowest plasma ^2^H‐alanine precursor enrichment. This could indicate a low compliance to the oral ^2^H_2_O intake for participant P01, despite self‐reported full compliance to the ^2^H_2_O intake.

### 3.2 Muscle deuterium enrichment and fractional synthesis rate

**FIGURE 3:**
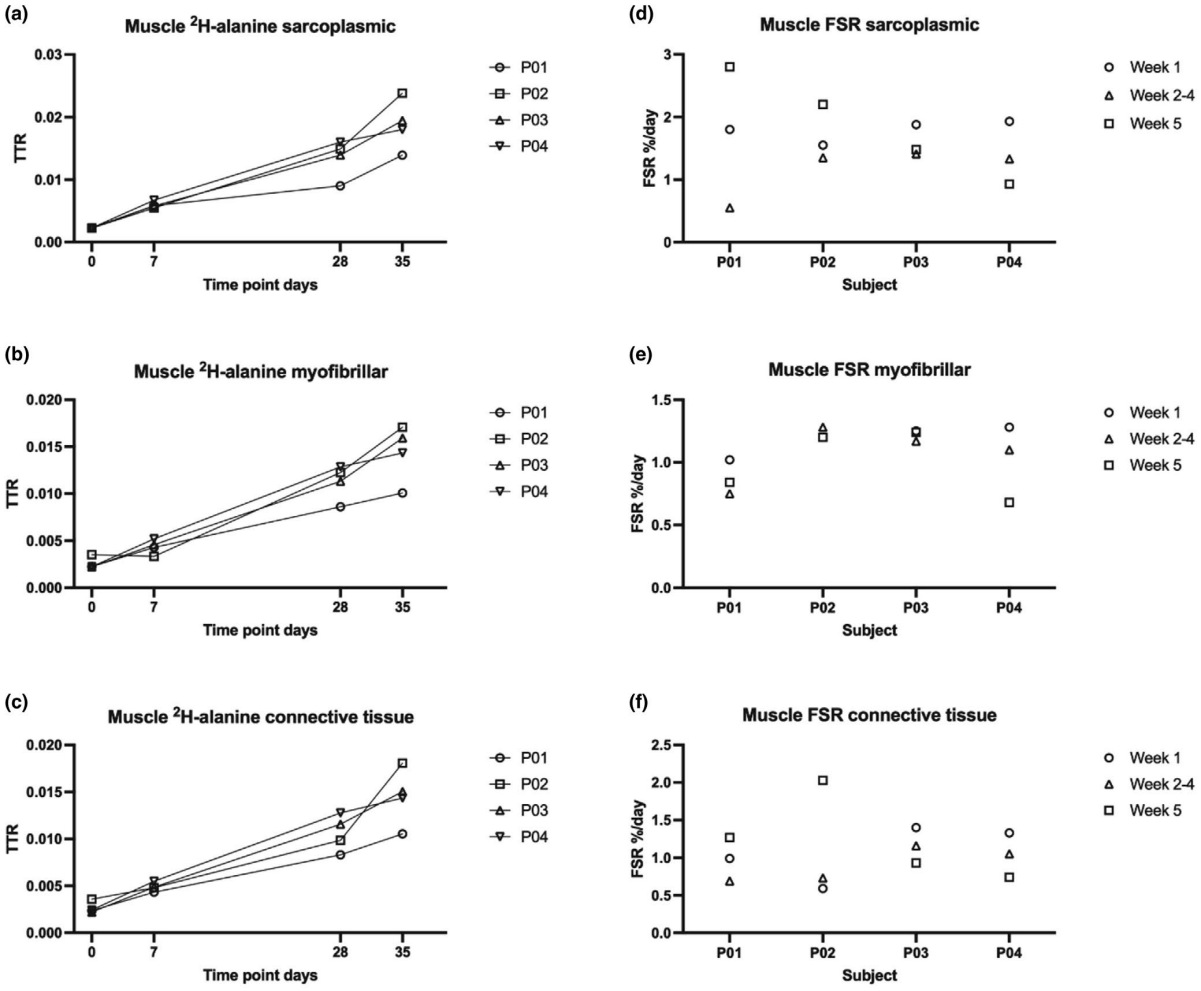
Muscle protein tracer‐to‐tracee enrichment of ^2^H‐alanine and fractional synthesis rate. Tracer‐to‐tracee (TTR) expression of ^2^H‐alanine in (a) sarcoplasmic, (b) myofibrillar, and (c) connective tissue fractions from biopsies taken on Day 0, 7, 28, and 35, respectively. Fractional synthesis rate (FSR) in % × day^−1^ is shown for the (d) sarcoplasmic, (e) myofibrillar, and (f) connective tissue fraction in Week 1, Week 2–4, and Week 5, respectively, for each of the four participants.

The tracer ^2^H‐alanine incorporation in muscle proteins was linear in all mixed fractions (sarcoplasmic, myofibrillar, and connective tissue) in the first 28 days (Figure 3a–c). From Day 28 to Day 35, the ^2^H‐alanine incorporation occurred at a higher rate for participants P02 and P03 in accordance with the elevated level of ^2^H_2_O precursor. The protein FSR in sarcoplasmic, myofibrillar, and connective tissue fractions from participants P02 and P03 was similar between the three measurement periods: Week 1, Week 2–4, and week 5 (Figure 3d–f). The lowest variation between measurement periods was in the myofibrillar fraction, whereas in participant P02, the FSR measured in the connective tissue fraction was much higher during Week 5 compared to Week 1 and Week 2–4.

In participant P04 (no step increment in plasma ^2^H‐alanine precursor enrichment during Week 5) the protein FSR in the sarcoplasmic, myofibrillar, and connective tissue fractions was lower during Day 28 to Day 35, compared to the period from Day 0 to 28 (Figure 3d–f).

Collectively, the lower rate of incorporation and lower FSR of protein in week 5 could indicate that a rise‐to‐plateau phase occurred in week 5 for participant P04, affecting the synthesis rate outcome. Therefore, measuring protein synthesis rates of mixed muscle fractions using amino acid (i.e. alanine) analysis beyond a 4‐week steady‐state deuterium labeling period might not be advisable. The same rise‐to‐plateau effect would have been expected in participant P01, that was supposed to follow the same pattern of ^2^H_2_O intake as participant P04. Of all four participants, participant P01 had the lowest increase in muscle protein ^2^H‐alanine incorporation. It is not possible to determine whether this was due to a habitual lower protein turnover or the aforementioned concern regarding compliance with ^2^H_2_O intake. In any case, with the lower rise in ^2^H‐alanine incorporation the rise‐to‐plateau effect was not seen in participant P01 in any of the muscle protein fractions.

### 3.3 Muscle ^15^N‐alanine enrichment and muscle protein breakdown

**FIGURE 4:**
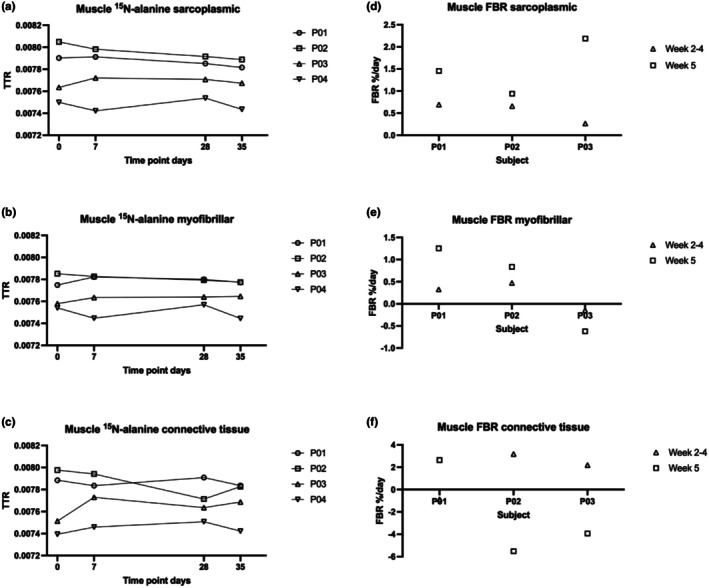
Muscle protein tracer‐to‐tracee enrichment of ^15^N‐alanine and fractional breakdown rate. Tracer‐to‐tracee (TTR) expression of ^15^N‐alanine in (a) sarcoplasmic, (b) myofibrillar, and (c) connective tissue fraction from biopsies taken on Day 0, 7, 28, and 35, respectively, for participants P01, P02, P03, and P04. Fractional breakdown rate (FBR) in % × day^−1^ is shown for the (d) sarcoplasmic, (e) myofibrillar, and (f) connective tissue fraction in Week 1, Week 2–4, and Week 5, respectively, for participants P01, P02, and P03.

^2^H_8_‐phenylalanine tracer was infused in participants P01 and P03 on Day −3 as a negative control for the measurement of protein breakdown. As expected, the muscle protein ^2^H_8_‐phenylalanine did not decay as the tracer was expected to recirculate (Figure S2). Interestingly, the muscle protein ^2^H_8_‐phenylalanine enrichment also increased in participants P02 and P04, who did not receive a ^2^H_8_‐phenylalanine infusion. This most likely occurred as a transamination of ^2^H_7_‐phenylalanine to ^2^H_8_‐phenylalanine due to the ^2^H_2_O administration to the participants.

On Day −3, prior to the measurement period, a ^15^N‐alanine flood was given to participant P01, P02, and P03 to achieve incorporation into muscle proteins. Plasma ^15^N‐alanine enrichment immediately increased after the infusion and had returned to baseline at Day 0 (Figure S1). Therefore, no further protein incorporation of ^15^N‐alanine was expected beyond this time point, which should enable measurement of muscle protein breakdown by following the decay in protein enrichment of ^15^N‐alanine. However, during the initial 7 days, a further incorporation of ^15^N‐alanine into sarcoplasmic and myofibrillar muscle proteins for participant P02 and P03 was seen (Figure 4a,b). Despite that plasma ^15^N‐alanine enrichments had returned to baseline, it could be speculated that intracellular free ^15^N‐alanine remained elevated during the first week. Therefore, the fractional breakdown rate (FBR) was only calculated during Weeks 2–4 and Week 5. The greatest variation in the breakdown rate was seen for participant P03, who received half the dose of ^15^N‐alanine on Day −3 compared to participant P01 and P02 (Figure 4d–f). Even for participant P01 and P02 the signal intensity during the mass spectrometry analysis was low. This was especially the case for the connective tissue fraction, which resulted in the greatest variation and several negative values (Figure 4f). Therefore, in future development of the method, it would be advisable to increase the tracer dose or use a tracer, such as ^13^C_3_‐alanine, that affords more sensitive measurements. Despite the variation in the breakdown rates of the sarcoplasmic and myofibrillar fractions and the need to increase sensitivity for future applications, the calculated FBR was comparable to the FSR, as is expected in a steady state situation indicating a potential feasibility in the approach.

### 3.4 Dynamic proteome profiling

Label‐free quantitation of protein abundances encompassed 1805 proteins that were detected in all samples (i.e. no missing data) at each experimental time point (Days 0, 7, 28 and 35) in all *n* = 4 participants. Protein abundance profiles exhibited high degrees of similarity (Pearson rho>0.82 across all participants and all time points).

**FIGURE 5:**
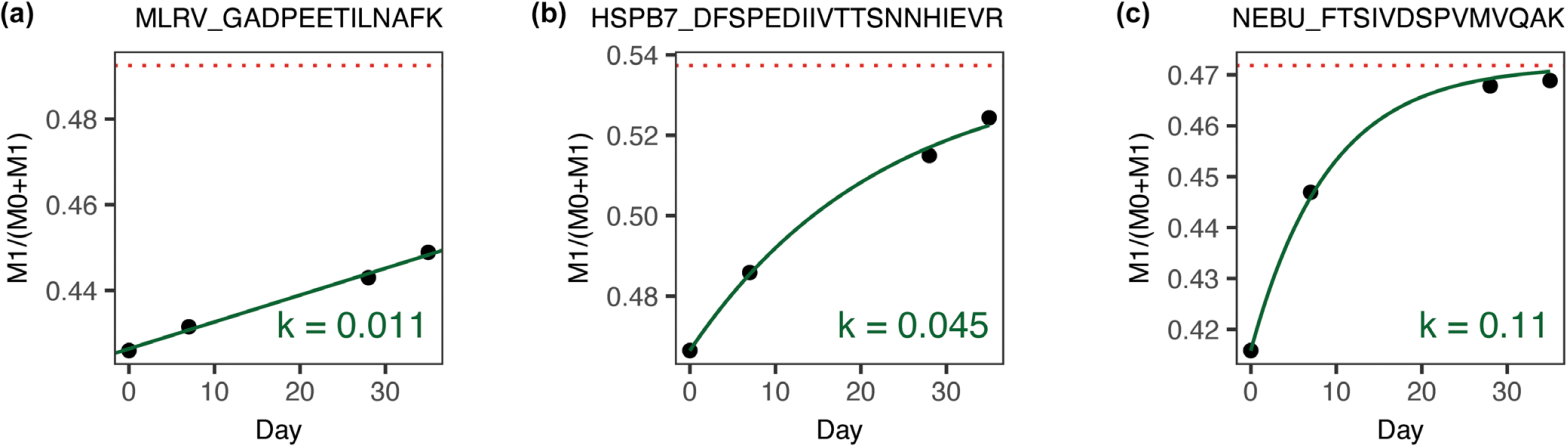
Representative labeling curves from low‐, medium‐ and high‐ turnover rate proteins. Asymptotic regression of peptide mass isotopomer ratio data in participant P04 that received only ^2^H_2_O and maintained a consistent body water enrichment of 0.855% throughout the 35‐day experimental period. Rate constants (K) of deuterium incorporation into muscle protein peptides were calculated by asymptotic regression curves to mass isotopomer data collected at Days 0, 7, 28 and 35 using the Levenberg–Marquardt ‘damped’ nonlinear least squares algorithm. Peptides from 3 proteins with different fractional turnover rates (FTR; FTR (%/d) = K *100), including: (a) Myosin regulatory light chain, slow/ventricular (MLRV), FTR = 1.1%/d; (b) Heat shock protein beta 7 (HSPB7), FTR = 4.5%/d; and (c) Nebulin (NEBU), FTR = 11%/d. Horizontal red dotted lines represent predicted plateau values of M1/(M0 + M1) ratio based on precursor enrichment and number of exchangeable H‐D sites in each peptide. Panel titles represent UniProt protein identifiers and amino acid sequence (single‐letter code) of each peptide.

**FIGURE 6:**
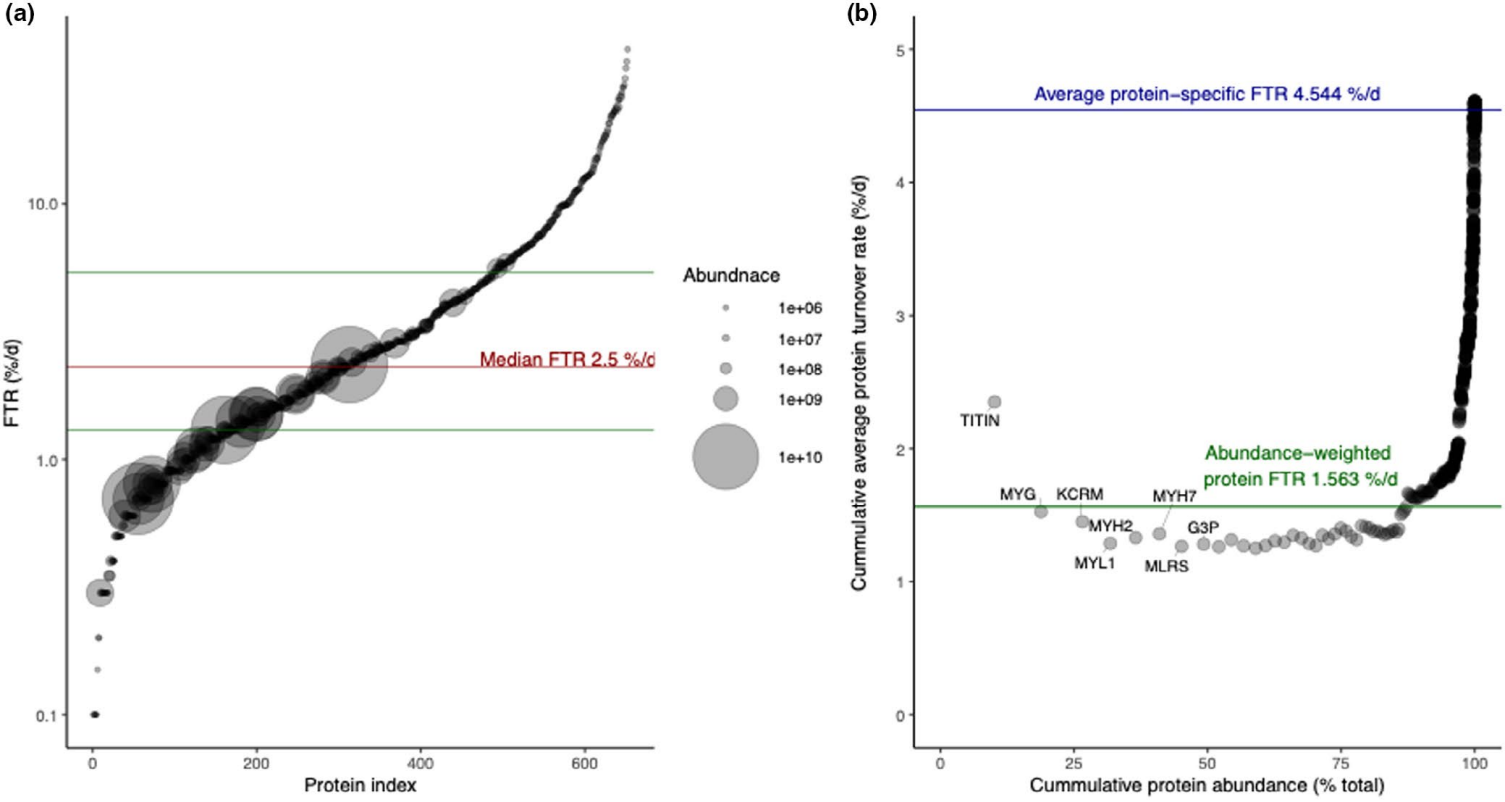
Contribution of protein‐specific turnover rates to muscle mixed‐protein average turnover. Turnover rate data for 652 proteins based on asymptotic regression on 3698 peptides from samples collected at Day 0 (unlabelled) and after 7 days, 28 days and 35 days of deuterium oxide labeling in vivo in participant P04 only. FTR values above 49.5%/d were excluded, based on the expectation these proteins would have been fully labeled before the first sampling point at Day 7. (a) Distribution of protein‐specific fractional turnover rates (FTR) ranked from lowest (0.1%/d; histone H2B type 1‐C) to highest (40.3%/d; collagen alpha‐1 XV, COFA1) rate of turnover. Data point size represents the relative abundance of each protein measured by label‐free quantitation. Horizontal lines denote the median (red), 1st and 3rd quartiles (green) of protein‐specific FTR (2.5%/d IQR = 1.3–5.4%/d. (b) The cumulative effect of increasing number of proteins studied beginning from the most abundant protein, titin (TTN; 2.35%/d). The top 50 most abundant proteins in skeletal muscle account for more than 85% of the total protein content and have relatively low rates of turnover, for example, Type I myosin heavy chain (MYH7) = 1.5%/d. Mean ± SD of protein‐specific fractional turnover rates was 4.54 ± 5.68%/d, whereas the abundance weighted average turnover of muscle proteins was 1.56%/d.

Participant P04 was not infused with stable isotope labeled amino acid tracers and received only ^2^H_2_O at a daily dose which resulted in a consistent body water enrichment of 0.855% across the 35‐day study period. Therefore, a reference dataset of protein‐specific turnover rates was generated by asymptotic regression on peptide data collected from unlabelled muscle (Day 0) and muscle collected after 7, 28 and 35 days of deuterium oxide consumption in participant P04 only. Asymptotic regression on only 4 time points can be challenging, and a stringent (RMSE < 10% error) filter was applied to select successfully fitted curves (Figure 5) for 3698 peptides from 652 proteins. The FTR of human muscle proteins ranged from 0.1%/d to 40.3%/d, mean = 4.54%/d, SD = 5.68%/d. Median = 2.5%/d IQR = 1.3–5.4%/d. The human muscle proteome included a small number of highly abundant myofibrillar proteins that had turnover rates below the median FTR of 2.5%/d (Figure 6a). When protein‐specific FTR data were weighted by the relative abundance of each protein, the aggregate turnover rate of mixed‐protein in human muscle was 1.56%/d. The abundance‐weighted mean FTR (1.56%/d) was less than the numerical average (4.54%/d) of protein specific FTR data because highly abundant myofibrillar proteins with relatively low (i.e. < 2.5%/d) individual rates of turnover made a greater contribution to the calculated mixed‐protein average (Figure 6b). For example, titin had a turnover rate of 2.35%/day and was the most abundant protein, accounting for ~10% of total muscle protein.

**FIGURE 7:**
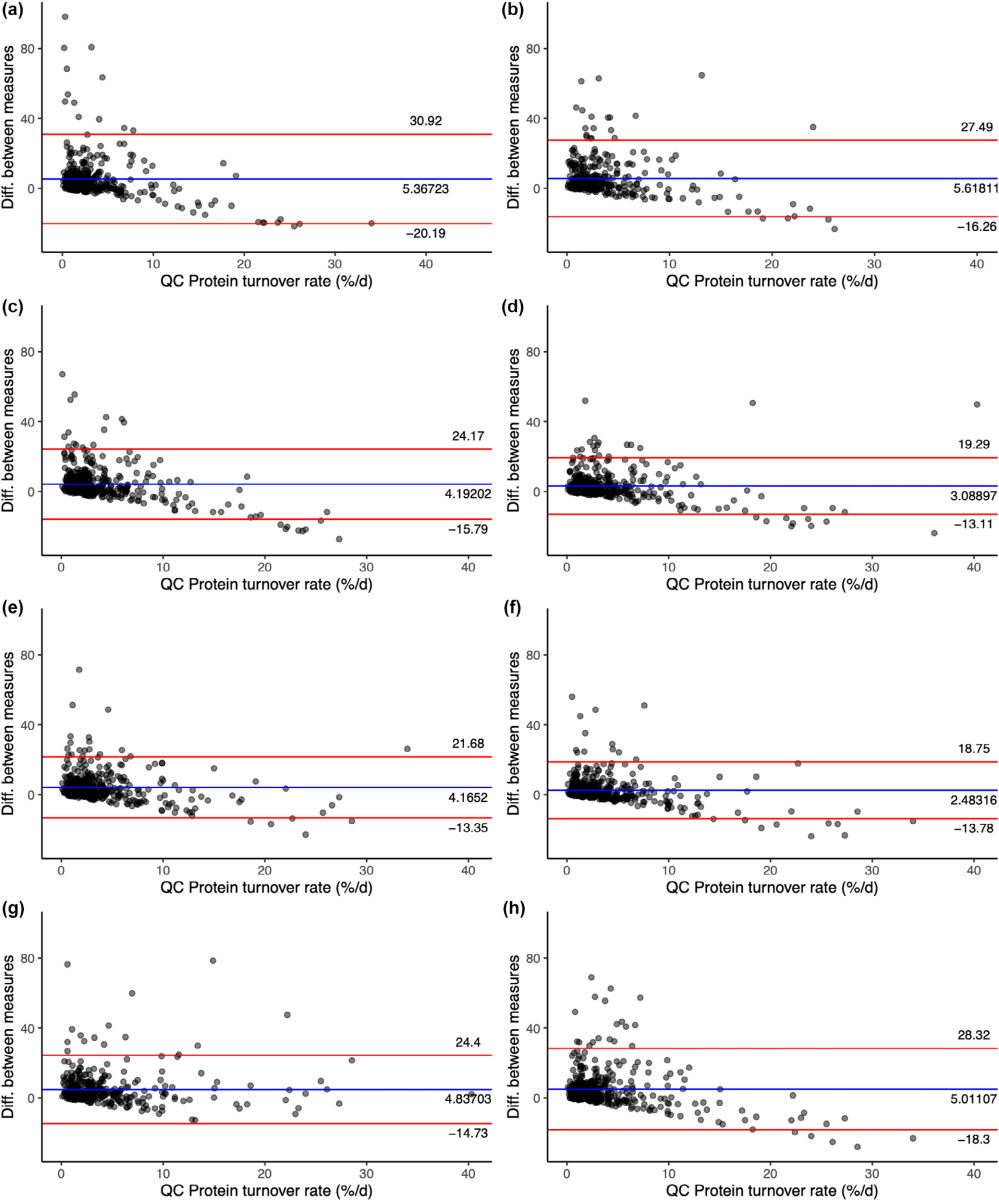
Comparison of NLS curve fit (QC) versus 2‐point calculation. QC turnover rate data (*x*‐axis) were acquired for 652 proteins based on non‐linear least squares (NLS) asymptotic regression on 3698 peptides in participant P04 only, and after excluding peptides with residual mean squared error < 10%. *Y*‐axis represents the difference in FSR value between a 2‐point calculation and QC asymptotic regression (2‐point calculation—QC asymptotic regression), and data are shown as mean with ±1.96 SD. Protein turnover rates calculated by 2‐point calculation between Day 0 and Day 7 (panels a, c, e and g) or between Day 28 and Day 35 (panels b, d, f and h) in participants P01 (a and b), P02 (C and D), P03 (e and f) and P04 (g and h). The 2‐point calculation tends to overestimate the turnover rate of low turnover‐rate proteins (i.e. 2‐point—QC is >0), whereas the turnover rate of proteins with higher rates of turnover may be overestimated by the 2‐point method (i.e. 2‐point—QC is <0). N.B. Use of NLS curve fitting as QC reference should be tempered by knowledge that good curve fitting is challenging on a time series of only 4 data points.

Two‐point calculations using data collected at the beginning and end of a labeling period are commonly used to measure muscle protein synthesis in human studies but this design cannot distinguish the different labeling kinetics of low and high turnover rate proteins. While DPP measurements are not without error, the ability to distinguish the different labeling kinetics of individual proteins (Figure 5) enables data generated by asymptotic regression to be used as a reference for the comparison of data derived from 2‐point calculations. Participant‐specific protein FTR data were generated for participants P01, P02, P03 and P04 during either the first week of labeling (Day 0–Day 7) or 5th week of labeling (Day 28–Day 35) based on 2‐point calculations using peptide data collected at the beginning and end of each 1‐week timespan, and were compared against the reference dataset generated from asymptotic regression data of participant P04 (Figure 7). A pattern emerged, indicating that the FTR of proteins with low turnover rates in the reference dataset tended to be overestimated when FTR data were generated by 2‐point calculation during either the first or fifth week of labeling. Conversely, the FTR of proteins with high rates of turnover in the reference dataset tended to be underestimated when FTR data were generated by 2‐point calculation during either the first or fifth week of labeling. The pattern (Figure 7) was indistinguishable between participants P02 and P03 that received a step increment in precursor enrichment during Week 5 (Figure 2) and participants P01 and P04 that maintained a consistent precursor enrichment throughout the 35‐day labeling period.

**FIGURE 8:**
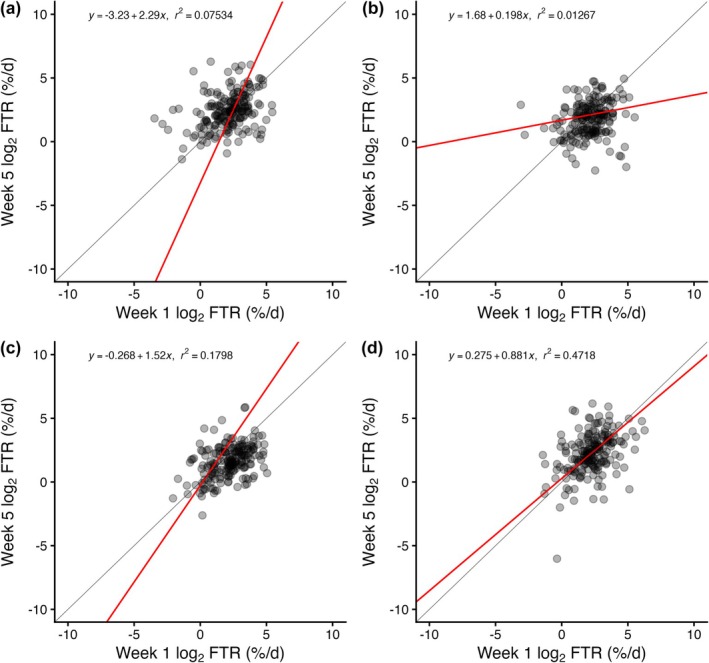
Comparison of FTR calculated by 2‐point method between early and late labeling period. Reduced major axis (RMA) regression of protein fractional turnover rates calculated during Week 1 (*x*‐axis) or week 5 (*y*‐axis) in participant P01 (a), P02 (b), P03 (c) and P04 (d). RMA 95% confidence intervals for the intercept suggest fixed bias in P01 (CI = −8.37 to −1.41) and P2 (CI = 1.23 to 2.12) but not P03 (CI = ‐1.23 to 0.33) and P04 (CI = −0.03 to 0.54). RMA 95% confidence intervals for the slope suggest proportional bias in P01 (CI = 1.51 to 4.47) and P03 (CI = 1.14 to 2.12) but not P02 (CI = −0.05 to 0.44) and P04 (CI = 0.75 to 1.02).

Within‐subject repeatability of protein FTR was assessed by reduced major axis (RMA) regression on 2‐point data calculated across either the first or fifth week of labeling (Figure 8). There were 195 common proteins that had FTR data from both the first and fifth week of labeling in all *n* = 4 participants. Participant P04 exhibited the strongest linear relationship (*R* ^2^ = 0.47) in protein‐specific FTR between week 1 and week 5 (Figure 8d). The median coefficient of variation (CV) between week 1 and week 5 FTR data was similar across participants (P01 = 29. 3%, P02 = 34.7%, P03 = 34.4% and P04 = 29.3%). RMA 95% confidence intervals suggested fixed bias was evident in participants P01 and P02 and proportional bias was evident in participants in P01 and P03.

Differences in repeatability may be due to interference effects of stable isotope labeled amino acid tracers or inconsistencies in ^2^H_2_O enrichment of body water (i.e. participant P01). Mass spectra of peptides enriched (>3 residues) in A|F|P residues were interrogated but no evidence of interference effects, e.g. significant disruption to expected mass isotopomer distribution in Day 0 samples was evident.

## 4 DISCUSSION

This pilot study was initiated to determine whether muscle protein synthesis measurements, either bulk fractions or single protein, are consistent over longer periods of ^2^H_2_O labeling, whether application of multiple tracers interferes with ^2^H_2_O labeling measurements, and whether simultaneous assessment of muscle protein synthesis and muscle protein breakdown is possible. We observed indications, that ^2^H_2_O labeling exceeding 4 weeks is increasingly vulnerable to tissue saturation (i.e. rise‐to‐plateau in label incorporation) when doing analyses on mixed‐protein muscle fractions (sarcoplasmic, myofibrillar and connective tissue) of human muscle. This appears likely to contribute to underestimation of bulk muscle protein FSR. This issue may potentially be mitigated by a step increment in ^2^H_2_O dose after the initial 4‐week labeling period. In contrast, for single protein analyses, the labeling period seems less critical during the 1‐ to 5‐week period, as the protein‐specific measurements enable the different labeling kinetics of individual proteins to be detected. Further this pilot study indicates a possibility of simultaneously measuring synthesis and breakdown, however for single protein analysis caution should be taken when adding multiple tracers, as this may confound the FSR data.

The appropriate period for ^2^H_2_O labeling studies depends on the protein turnover rate of the tissue. Human skeletal muscle has an average turnover rate of 1–2%/day and previous ^2^H_2_O studies (Crossland et al., 2022; Robinson et al., 2011; Wilkinson et al., 2014) have used labeling periods from 2 days to 8 weeks. However, the generally accepted, 1–2%, daily turnover rate of mixed protein in human muscle is the average of many individual proteins with different turnover rates and abundances (Camera et al., 2017). In the current work, one‐quarter of the >650 proteins studied had turnover rates above 5.4%/day (Figure 6a) and are therefore expected to approach a plateau in label enrichment within 4 weeks which affects FSR measurements calculated over, for example, 1 week compared to 4 weeks. Furthermore, the median turnover rate was 2.5%/ day (Figure 6a), which equates to a half‐life of 27.8 days. This suggests that the ^2^H‐alanine enrichment of the proteins studied might approach ~50% of the precursor plateau enrichment by Day 28. Muscle protein turnover is expected to be unchanged during the 5‐week study period as no changes to the diet or daily activity were made, and no changes in protein abundance were detected. Therefore, the influence of the higher turnover rate proteins could explain the non‐linear pattern of change in muscle protein labeling depicted in the sarcoplasmic, myofibrillar and connective tissue fractions in participant P04 (Figure 3a–c) that received a constant dose of ^2^H_2_O and exhibited a consistent level of precursor enrichment. Beyond Day 28, tissue enrichment was clearly non‐linear (i.e. rise‐to‐plateau effect) in participant P04, which resulted in substantially lower FSR values in week 5 (Figure 3d–e).

Participant P01 was also expected to have a plateau in tissue protein enrichment after 28 days ^2^H_2_O labeling, as for participant P04. But the measured variability in precursor enrichment in participant P01 likely accounts for the difference in result compared to P04. Participants P01 and P04 were each prescribed ^2^H_2_O with the aim of achieving a body water enrichment of 1% ^2^H_2_O throughout the 35‐day experimental period. However, only participant P04 exhibited a flattening of the tissue enrichment curve. This can be due to individual variability; however, it is also likely, as written in the results section, that this is due to missed ^2^H_2_O ingestions from participant P01. In the plasma enrichment (Figure 2), participant P01 has a high variability in plasma ^2^H‐alanine enrichment, which could indicate that some of the daily ^2^H_2_O doses were missed. Likewise, the tissue enrichment in participant P01 is consistently lower than the other participants, even in the first 28 days, where all participants were receiving the same relative ^2^H_2_O dose. Although ^2^H_2_O is administered orally, the delivery should be treated with a level of rigor similar to that used for amino‐acid infusions. Our findings highlight that supervised dosing or in‐clinic administration on more days may be required to ensure true steady‐state precursor behavior in future studies. Furthermore, frequent measurements of precursor enrichment—obtained in this study through repeated blood sampling—are important for calculating a weighted average that accurately reflects potential variability in precursor enrichment. In retrospect, the variability in P01 underscores that unsupervised oral dosing introduces a real risk of precursor instability. We therefore recommend that future long‐term ^2^H_2_O protocols incorporate tighter monitoring or supervised administration during the entire labeling period as far as feasible. Nonetheless, whether it is this specific case of possible dosing inconsistency, biological variability, or other unexplained precursor instability, based on the high number of single proteins with an FSR above 2.5%/day caution might be advisable if the labeling period exceeds 28 days. During interventions such as structured resistance exercise, an increase in muscle protein synthesis rate would be expected (Lim et al., 2022; Witard et al., 2014), and the rise‐to‐plateau in protein enrichment would accordingly occur earlier. Despite these challenges, bulk skeletal muscle protein turnover could still be studied over longer periods, as suggested by participant P02 and P03, who increased the ^2^H_2_O dose after 28 days, aiming at a body water ^2^H_2_O enrichment of 2% in the last week.

The possibility that a higher synthesis may lead to faster rise‐to‐plateau and consequently underestimated FSR values with higher variability is illustrated in Figure 5, showing that the single proteins with higher FSR are more likely to exhibit saturation within the 5‐week period. Such differences in protein‐specific FSR are hidden from, but nevertheless impact, the accuracy of mixed‐protein FSR measurements. In contrast, protein‐specific measurements enable proteins reaching the enrichment plateau to be identified and, therefore, are less affected by limitations due to rise‐to‐plateau in protein labeling. However, this is only empirically evident when a time series of samples (i.e. in this case 0, 7, 28 and 35 days) is analyzed. In the current work, data generated by non‐linear curve fitting across 4 time points in participant P04 was used as a reference to illustrate the limitations of rate constants calculated from 2 time points that assume, but do not measure, rise‐to‐plateau kinetics. The reference values we report are specific to participant P04 and future work encompassing greater numbers of participants and sampling times would be necessary to generate population‐level reference data.

Figure 8 suggests that participant P04 has the best line of agreement between protein FSR measured during Week 1 and Week 5, indicating that the calculation of individual protein FSR was not significantly impacted by the rise‐to‐plateau effect that was evident in mixed‐protein FSR data of the sarcoplasmic, myofibrillar, and connective tissue fractions of this participant. The greater discrepancies observed between individual protein FSR calculated during week 1 compared to week 5 in participants P01, P02, and P03 (Figure 8) may be due to confounding effects of the additional tracers received by these participants. Therefore, although we could not detect obvious differences in peptide mass isotopomer profile in these pilot data, the addition of other amino acid tracers seems unfavorable when measuring single protein turnover with ^2^H_2_O. In particular, tracers, such as ^15^N‐proline, could introduce complexities in peptide mass isotopomer distributions, which are needed to assess deuterium incorporation for protein‐specific FSR calculations. For example, ^15^N‐labeled amino acids administered around the time of the baseline sample collection could alter peptide mass isotopomer patterns in a manner that is similar to the effect of deuterium incorporation. During the early period of the experiment, the effect of ^15^N labeled amino acids on the peptide mass isotopomer profile would be diminishing and this would co‐occur with the rise in RIA due to biosynthetic labeling of proteins with deuterium – essentially the baseline could be artificially raised and this would be expected to result in an underestimation of protein turnover rates by deuterium oxide labeling. Therefore, caution is warranted and higher doses or amino acid tracer administration closer to the baseline biopsy could pose a greater interference risk.

The decision to include multiple tracers was done to assess protein breakdown. Since skeletal muscle turnover is the sum of synthesis and breakdown, simultaneous measurement of both processes is often desirable. We introduced the ^15^N‐alanine tracer on Day −3 (3 days before the ^2^H_2_O administration and the first biopsy) for participants P01, P02, and P03 as a single bolus flood. The aim was to introduce the alanine tracer early enough to achieve tissue protein labeling with the ^15^N‐alanine and at the same time ensure that the free ^15^N‐alanine enrichment returned to baseline before commencing muscle protein breakdown measurements. This occurs relatively fast due to the high rate of transamination of alanine. However, while the plasma enrichment of ^15^N‐alanine did return to baseline, the tissue enrichments seemed to increase in the first week, indicating a potential further incorporation of ^15^N‐alanine beyond Day 0. This made it impossible to follow the tissue disappearance of ^15^N‐alanine to assess breakdown in week 1. In the later time points in the bulk tissue analysis of the sarcoplasmic, myofibrillar, and connective tissue fraction (Figure 4), the clearest decline in ^15^N‐alanine protein enrichment is seen in participants P01 and P02 receiving the highest doses of ^15^N‐alanine. Notably, under the given study conditions, a net balance between synthesis and breakdown is assumed, and in participants P01 and P02, the fractional breakdown rate is to some degree comparable to the level of fractional synthesis rate, indicating a potential feasibility of the method. Nevertheless, the variability is high when measuring the FBR by pre‐labeling with a ^15^N‐alanine flood. Therefore, the approach remains experimental and requires further optimization by increasing the tracer sensitivity. This can be addressed in one of two ways: (1) the tissue tracer enrichment needs to be higher before following the tracer disappearance from the tissue and/or (2) the sensitivity of the tracer should be better by applying a tracer with more labels, making the tracer more easily distinguishable from the natural isotopic background. The latter may be achieved by choosing a tracer with more labels, which are distinct from other tracers used, making it easier to detect by mass spectrometry.

### 4.1 Future considerations

As the field of proteomics continues to advance, we can bridge the gap between traditional stable isotope tracing methods and proteomic techniques. This will increase our understanding of tissue plasticity, regeneration, and metabolic adaptations in health and disease. However, to ensure the robustness and accuracy of muscle protein turnover studies, future research should address the complexities associated with long periods of ^2^H_2_O labeling when assessing protein turnover with single proteins with vastly different turnover rates. Further, focus should be on optimizing tracer dosing strategies and carefully selecting amino acid tracers to minimize confounding effects when using multiple tracers to do analyses on single proteins. With this, using the enhanced sensitivity in tracer analysis techniques and advanced proteomic approaches we can generate more comprehensive insights into muscle protein turnover dynamics, facilitating a deeper understanding of tissue plasticity and metabolic adaptations.

This pilot study identified key challenges and considerations when performing studies with long periods of ^2^H_2_O labeling and when utilizing multiple tracers for assessing muscle protein turnover. However, a major limitation of the present pilot study is the very small sample size (*n* = 4) and the deliberately heterogeneous tracer setup across participants. These design choices prevent meaningful statistical testing and strongly limit generalisability. Moreover, the lack of strict dietary and physical activity control introduces additional biological variability. We also observed inter‐individual fluctuations in precursor enrichment, which further illustrates the challenges of applying long‐term ^2^H_2_O labeling under free‐living conditions. Consequently, the findings should be interpreted as mechanistic and methodological observations rather than population‐level physiological effects. Controlled studies on a larger scale would be needed for further optimization of measuring integrated FBR, and methodological refinements and meticulous experimental design are needed to enhance the reliability and interpretability of single muscle protein turnover data.

## 5 CONCLUSION

When doing bulk protein synthesis measures, labeling periods exceeding 4 weeks might underestimate the fractional synthesis rate, and the data will reflect synthesis of different pools of proteins. Increasing the ^2^H‐alanine precursor enrichment could extend the measurement period beyond 4 weeks.

Conversely, ^2^H_2_O labeling for up to 5 weeks seems feasible for single protein analysis when accounting for the rise‐to‐plateau for each individual protein. Notably, mixing tracers can confound measurements of single protein turnover and should be carefully considered during study design, and at the very least, reported in publications.

Measuring muscle protein breakdown with a labeled alanine tracer simultaneously with ^2^H_2_O determined muscle protein synthesis appears potentially feasible, though additional methodological improvements are required.

Overall, these conclusions should be viewed as preliminary and reflective of the exploratory pilot nature of the study. Larger controlled cohorts will be necessary to confirm these patterns.

## AUTHOR CONTRIBUTIONS

**Grith Højfeldt:** Conceptualization; data curation; investigation. **Bjørk Wulff Helge:** Data curation; investigation. **Gerrit van Hall:** Conceptualization; formal analysis; methodology; validation. **Peter Schjerling:** Conceptualization; formal analysis. **Michael Kjær:** Conceptualization; funding acquisition. **Abigail L. Mackey:** Conceptualization; funding acquisition. **Jatin G. Burniston:** Conceptualization; data curation; formal analysis; methodology; validation. **Jakob Agergaard:** Conceptualization; data curation; formal analysis; investigation; methodology; project administration; visualization.

## FUNDING INFORMATION

The BRIDGE—Translational Excellence Programme (bridge.ku.dk) at the Faculty of Health and Medical Sciences, University of Copenhagen, funded by the Novo Nordisk Foundation (Grant ID: NNF20SA0064340 to G.H.) and Independent Research Fund Denmark (Grant ID: 10.46540/3101‐00063B to A.L.M.).

## CONFLICT OF INTEREST STATEMENT

None of the authors has any conflicts of interest.

## ETHICS STATEMENT

All participants provided written consent prior to enrolling in the study, and the study was conducted in line with the Declaration of Helsinki. The study was approved by Ethics Committee of Copenhagen and Frederiksberg (H‐21025129).

## Supporting information


**Figure S1.** Plasma ^15^N‐alanine enrichment in mole percent excess (MPE) on Day −3, before and 2 h after bolus injection of ^15^N‐alanine, and on Day 0 for Participants P01, P02, and P03.
**Figure S2.** Muscle protein ^2^H_8_‐phenylalanine tracer‐to‐tracee (TTR) expression in (a) sarcoplasmic, (b) myofibrillar, and (c) connective tissue fractions on Day 0, 7, 28, and 35, respectively, for Participants P01, P02, P03, and P04. Note, Participants P02 and P04 did not receive a bolus injection of ^2^H_8_‐phenylalanine on Day −3.

## Data Availability

The mass spectrometry proteomics data have been deposited to the ProteomeXchange Consortium (http://proteomecentral.proteomexchange.org) via the PRIDE partner repository (Perez‐Riverol et al., 2019) under the dataset identifier PXD078552 (https://www.ebi.ac.uk/pride/archive/projects/PXD078552).
