# Supplementary material for: 2H2O labeling methods for bulk and single muscle protein synthesis measures, and measures of integrated muscle protein synthesis and breakdown rates: A pilot study
Source: Physiol Rep. 2026 Jul 9;14(13):e71013. doi: 10.14814/phy2.71013 (PMC13347171; doi:10.14814/phy2.71013)
Supplement: Supplementary file 1 — Figure S1. Plasma 15N‐alanine enrichment in mole percent excess (MPE) on Day −3, before and 2 h after bolus injection of 15N‐alanine, and on Day 0 for Participants P01, P02, and P03. Figure S2. Muscle protein 2H8‐phenylalanine tracer‐to‐tracee (TTR) expression in (a) sarcoplasmic, (b) myofibrillar, and (c) connective tissue fractions on Day 0, 7, 28, and 35, respectively, for Participants P01, P02, P03, and P04. Note, Participants P02 and P04 did not receive a bolus injection of 2H8‐phenylalanine on Day −3. [file PHY2-14-e71013-s001.docx]

# Supplementary data


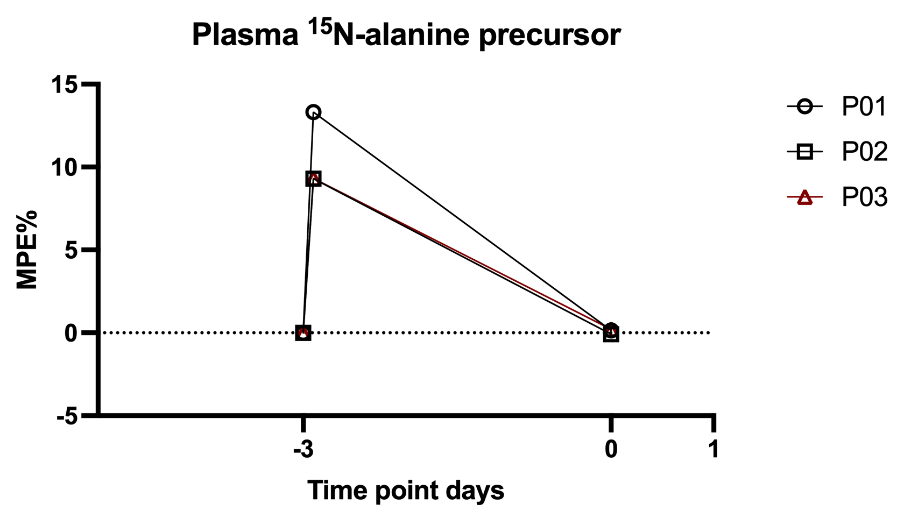


**Supplemental figure 1**

Plasma ^15^N-alanine enrichment in mole percent excess (MPE) on day -3, before and 2 hours after bolus injection of ^15^N-alanine, and on day 0 for Participants P01, P02, and P03.


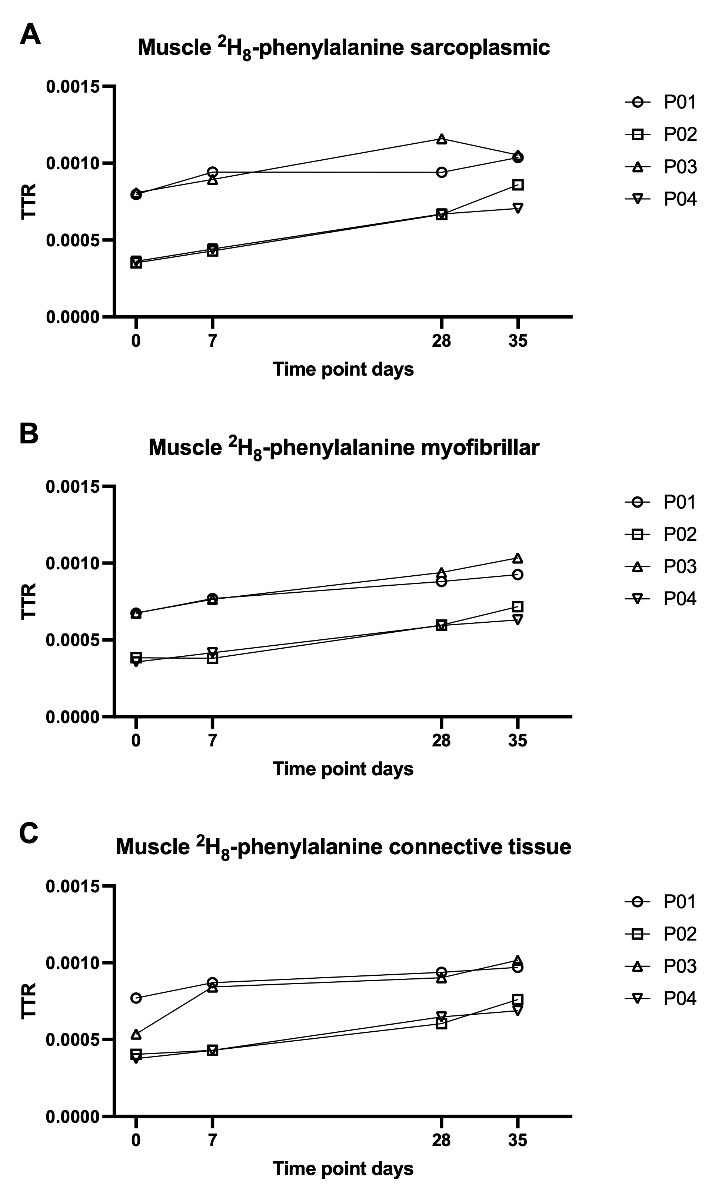


**Supplemental figure 2**

Muscle protein ^2^H_8_-phenylalanine tracer-to-tracee (TTR) expression in A) sarcoplasmic, B) myofibrillar, and C) connective tissue fractions on day 0, 7, 28, and 35, respectively, for Participants P01, P02, P03, and P04. Note, Participants P02 and P04 did not receive a bolus injection of ^2^H_8_-phenylalanine on day -3.
